# Obesity, leptin, and deregulation of microRNA in lipid metabolisms: their contribution to breast cancer prognosis

**DOI:** 10.1186/s13098-020-00621-4

**Published:** 2021-01-22

**Authors:** Kartika W. Taroeno-Hariadi, Mardiah S. Hardianti, Hemi Sinorita, Teguh Aryandono

**Affiliations:** 1 Division of Hematology and Medical Oncology, Department of Internal Medicine, Faculty of Medicine, Public Health, and Nursing, Universitas Gadjah Mada, Farmako Street, Sekip Utara, Yogyakarta, 55281 Indonesia; 2 Division of Endocrinology, Department of Internal Medicine, Faculty of Medicine, Public Health, and Nursing, Universitas Gadjah Mada, Yogyakarta, Indonesia; 3 Division of Surgical Oncology, Department of Surgery, Faculty of Medicine, Public Health, and Nursing, Universitas Gadjah Mada, Yogyakarta, Indonesia

**Keywords:** Obesity, Lipid metabolism, microRNA, Breast cancer, Prognosis

## Abstract

Obesity and Metabolic Syndrome have been associated with cardiovascular, diabetes and cancer incidence. Obesity is a state of inflammation. There are cross-talks between adipocyte, adipokines, pro-inflammatory cytokines, insulin, leptin, and other growth factors to initiate signals for proliferation, anti-apoptosis, and angiogenesis. Those networks lead to cancer initiation, promotion, progression, and metastasis. Post menopause women with breast cancer commonly have overweight, obesity, and metabolic syndrome, which are previously reported as conditions to be associated with breast cancer prognosis. MicroRNAs (miRNAs), small non-coding RNA that regulate gene expression, are known to play important roles either in metabolic or carcinogenesis process in patients with breast cancer. Some miRNAs expressions are deregulated in persons either with obesity, breast cancer, or breast cancer with co-morbid obesity. This literature review aimed at reviewing recent publications on the role of obesity, leptin, and microRNA deregulation in adverse prognosis of breast cancer. Understanding the influence of deregulated miRNAs and their target genes in patients with breast cancer and obesity will direct more studies to explore the potential prognostic role of obesity in breast cancer from epigenetic points of view.

## Background

Breast cancer has become the most common malignancy in women all over the world, with estimated 2,088,089 new cases and 626,679 deaths in 2018 [1]. Due to its high incidence and mortality rate, various prevention strategies have been developed in risk identification and stratification, screening, early detection and diagnosis, personalized treatment, and identification of biomarkers.

Many studies proved the importance of metabolic biomarkers in cancer risk and prognosis, including in breast cancer [2–7]. Metabolic biomarkers are used clinically in predicting the responses to certain cancer treatments, assessing drug toxicity, monitoring efficacy, and evaluating pharmacodynamics. Identification of metabolic markers has been explored and widely applied since the last two decades. Some of the major concerns involve the comorbidities of obesity and Metabolic Syndrome, which are known as risk factors for cardiovascular disease, diabetes, and for cancer as well [8]. Obesity increases the risk for breast cancer and research found that 46.2% of patients with breast cancer are obese [8]. Some studies also reported that obesity influenced the prognosis of breast cancer [8].

Many theories have been raised to explain the role of obesity in cancer. It is well-established that there is a crosstalk between tumor microenvironment, adipocytes, adipokines, estrogen, and proliferation of cancer cells [9]. Cancer cells also influence surrounding tissue to support their growth, cell cycle, invasion, and migration [10]. Studies on leptin, which is a protein hormone secreted by adipocytes, and identified as a candidate biomarker for breast cancer progression, have shown unconvincing results, and none of the hypotheses concerning the role of leptin or other adipokines in breast cancer progression have been totally conclusive.

MicroRNA (miRNA) is a non-coding, small molecule RNA that has the function to modulate expression of many protein coding-genes, thereby contributing to both physiologic and pathologic processes. The roles of miRNA as a regulator for both the metabolic process and carcinogenesis in breast cancer are abundantly reported [11, 12].

This literature review aimed to reviewing recent developments and publications concerning the role of obesity, leptin, and miRNA deregulation in adverse prognosis of breast cancer. We reviewed all publication from PubMed database using the terms ((“breast neoplasms”[MeSH Terms] AND (“leptin”[MeSH Terms] AND (“obesity”[MeSH Terms] OR (“metabolic syndrome”[MeSH Terms] AND (“micrornas”[MeSH Terms]. We consider it is important to explore substantial available data, analyze, and unfolded the complexity of several miRNAs that regulate the expression of certain genes involved in interaction between metabolic and mitogenic pathway in breast cancer.

## Obesity and metabolic syndrome increase breast cancer risk and progression

Obesity related hyperinsulinism and high circulating estrogen levels may explain the association between adipocytes and breast cancer cells. In post-menopausal women, adipocytes become the main source of aromatase contributing to the increased number of adipocytes in obese women, in which the more the increase, the greater the androgen aromatization into the hormone estrogen. In obese post-menopausal women, there are increased levels of plasma insulin and insulin-like growth factor-1 (IGF-1) that have mitogenic effects on breast cancer cells [13]. Obesity and excess fat present as chronic inflammation, insulin resistance, adipokines imbalance, and increased estrogen signaling [14].

Epidemiology studies show there are associations between obesity-related inflammation and breast cancer incidence, prognosis, and mortality rate. Obesity, defined as increased Body Mass Index (BMI) ≥ 30, is related to breast cancer risk, with relative risk (RR) ranges from 1.5–2.5 [15]. Increased risk of breast cancer in post-menopausal women with increased BMI are largely caused by hormonal increases of free-estradiol [16, 17]. Patient characteristics such as post menopause, obese, unhealthy metabolism, and central obesity in women have been shown to increase the risk for developing breast cancer. [2, 6, 14, 18].

Metabolic Syndrome, defined as having three or more metabolic components (obesity or increased waist circumference, hyperglycemia, hypertriglyceridemia, low-HDL, or hypertension) [19, 20], is also associated with breast cancer risk. The hazard ratio of Metabolic Syndrome in breast cancer risk in Japan is reported to be 2.87 (CI 1.67–4.94), and the hazard ratio for post-menopausal breast cancer is 6.73 (CI 2.93–25.43) [21].

Obesity is also associated with poorer survival of breast cancer [22, 23]. High BMI has been proven to be an independent prognostic factor in triple negative breast cancer (TNBC) as well as in luminal type breast cancer [16, 24]. Additionally, higher Waist-to Hip (WTH) ratio is a poorer prognostic factor in luminal type breast cancer [25]. Chan et al. reported a meta-analysis of 82 studies which involved more than 200,000 breast cancer patients [26]. This study reported that obese patients have poorer total mortality (HR = 1.41, 95% CI 1.29–1.53) and poorer breast cancer-specific mortality (HR = 1.35, 95% CI 1.24–1.47) compared to normal weight patients [26].

## Estrogen and breast cancer

Estrogen is a steroid hormone that have several physiological functions such as: regulation of menstrual cycle and reproduction, development of breast tissue and sexual organ, bone density, brain function, energy balance, cholesterol mobilization and lipid homeostasis, β-cell function survival, insulin sensitivity, and control of inflammation [27]. Estrogen is widely known to have an important role in promoting and maintaining breast cancer tissue. In most breast cancer, estradiol (E2), the predominant type of estrogen in circulation, and Estrogen Receptor (ERα or ERβ) complex (E2-ER complex) are involved in malignant transformation. E2-ER complex activates transcriptional processes and/or signaling events to control gene expression. These actions are mediated through direct binding to specific sequences in gene promoters such as Estrogen Response Element (ERE) and other transcription factors (TFs) inside nucleus (direct genomic pathway) or by mechanisms that do not involved direct binding to DNA (indirect genomic pathway). Indirect genomic pathway involved action of E2 through membrane bound ER (G protein coupled Estrogen Receptor, GPCR1), which in turn recruited adaptor proteins interaction, second messenger production, cAMP regulation, and Mitogen Activated Protein Kinase (MAPK) and PI3K-Akt (Phosphatidyl Inositol-3 kinase/serine threonine kinase) pathway activation. These signaling cascades activation results in indirect changes of gene expression. Genes expression needed for cellular proliferation and growth functions are also activated by ER-ligand independent (estrogen independent) pathway through protein kinase signaling from growth factor receptors (IGFR, EGFR, HER) or by ER independent manner. There is crosstalk, and deregulation between ER, Insulin like Growth Factor Receptor (IGF-R), Growth Factors Receptors (EGFR, HER), and other RTK (Receptor Tyrosine Kinase) signaling to initiate and promote proliferation and metastasis of breast cancer cells. [28, 29].

## Adipose tissue, adipokines, and breast cancer

The precise mechanisms linking obesity and breast cancer remain unclear. One possible mechanism is the crosstalk between adipose tissue, microenvironment, and breast cancer cells. Adipose tissue may produce a group of polypeptide growth factors and cytokines such as adiponectin, leptin, Interleukin-6 (IL-6), Tumor Necrosis Factor-alpha (TNF-α), Plasminogen Activator Inhibitor-1 (PAI-1), and resistin that may underlie such association [20]. Current studies showed that in obese women the concentrations of circulating adipokines are elevated except for adiponectin, which is involved in glucose regulation and the metabolism of fat [30].

Recent publications have indicated that adipokines play an important role in the association between obesity and postmenopausal breast cancer [31]. In breast tissue there may be interactions between adipokines, estrogen, growth factor signaling, and hormones that create a microenvironment which promotes proliferation, growth, and survival of breast cancer cells [31, 32]. Recent research established that there is interaction between leptin and adiponectin signaling pathways in MCF-7 breast cancer cell lines, in which proliferation is induced by leptin and suppressed by adiponectin [33].

## Leptin and breast cancer

Leptin is a protein hormone produced by adipocytes, the placenta, and mammary epithelium. Leptin is a 16kD molecular weight protein encoded by obese genes. Leptin has several functions in controlling metabolic, reproductive, and immunologic processes, as well as angiogenesis, hematopoiesis, and lipid oxidation, and also acts as a pro-inflammatory factor. Plasma leptin level increases alongside with BMI [34–36].

Niu et al. [37] reported in their review and meta-analysis that leptin level plays a significant role in patients with breast cancer compared to healthy controls. Leptin enhances the proliferation of breast cancer cells by inhibiting the pro-apoptosis machinery, upregulating anti-apoptosis genes, modulating tumor microenvironment, and by increasing sensitivity to estrogen. Leptin in several studies of animal models is associated with breast cancer tumorigenesis. Leptin levels, both in circulating plasma or expression in breast cancer tissue, are reported to have association with breast cancer progression Leptin and leptin receptor are overexpressed in breast cancer tissue probably due to hypoxia, IGF, estradiol, and insulin overexposure [38].

## Mechanisms of obesity, metabolic syndrome, and deregulation of leptin in the breast cancer pathogenesis

There are cross-talks between obesity and carcinogenesis. They involve inflammatory pathways and are characterized by the dysregulation of metabolism. Obesity will increase aromatization of estrogen in adipose tissue, which in turn modulates sensitivity to insulin. In the process, leptin production is increased and as a result, a hyperinsulinemia condition occurs to modulate the mitogenic and anti-apoptotic effect.

Hyperinsulinemia is a condition where the cells decrease their sensitivity to insulin. Hyperinsulinemia can increase bioactivity of IGF-I, which involves binding to IGF-IR and a hybrid receptor of insulin receptor, isoform-A/IGF-I. Signals through these receptors may increase cellular proliferation via PI3K deregulation. PI3K will induce Akt and activate mTOR to stimulate protein synthesis, cell growth, and mitotic preparation. Dysregulation of mTOR is commonly found in various cancer including breast cancer. PTEN, a tumor suppressor gene, is often mutated or dysregulated so that proliferation signals through PI3K and IGF-I are uninhibited.

The stromal tumor microenvironment consists of matrix, fibroblast, vasculature, and immune cells play an important role in breast cancer carcinogenesis. Adipocytes are the main component of breast cancer’s stromal tissue. Many studies revealed an interaction between adipocytes and cancer cells, and their reciprocal adaptation will promote cancer progression. Adipocyte cells will induce cancer cells to proliferate, grow, migrate, and develop treatment resistance. Meanwhile, cancer cells will secrete paracrine and endocrine signals to mobilize metabolic substrates, especially free fatty acids, and to accumulate adipocyte cells around the tumor. Adipocyte cells will serve tumor cells by supporting them with metabolic substrates, lipid signal agonists, and also growth factors [9].

Accumulation of adipocytes will increase aromatase and in turn estrogen synthesis will be increased. Obesity, hyperinsulinemia, and increased IGF-I will decrease sex hormone binding globulin (SHBG) leading to increasing of estrogen bioavailability. Estrogen pathways are synergized with IGF-IR to activate Mitogen-Activated Protein Kinase (MAPK). Estrogen activates IGF-IR and Insulin Receptor Substrate (IRS-1 and IRS-2), which increases phosphorylation of IRS-1 and activation of MAPK [9].

Adiposity status is represented directly by leptin and its level in circulation is increased in obese or overweight patients. Leptin resistance is found in obesity and is caused by several mechanisms such as: defect access of leptin to its receptor (decrease receptor expression or impairment of post-receptor signaling including epigenetic process) [39], defect in the leptin blood–brain barrier, or weakening of leptin signaling due to inactivation of JAK-STAT pathway (via inhibition of suppressor of cytokine-signaling-3), endoplasmic reticulum stress, and inflammation [40]. Dysregulation of leptin signaling leads to more leptin secretion by adipocyte tissue.

Leptin acts on their receptors named leptin receptor Ob-R. Just as other cytokine receptors, the leptin receptor is a member of the cytokine I receptor superfamily. There are many isoforms of Ob-R, including: Ob-Ra, Ob-Rb, Ob-Rc, Ob-Rd, Ob-Re, and Ob-Rf. Ob-R a, c, d, and f have a short form of the cytoplasmic domain that activates MAPK pathways. The MAPK pathways, PI3K/phosphodiesterase 3B (PDE3B), and cyclic adenosine monophosphate (AMP) have roles in carcinogenesis. The full and long cytoplasmic domain is owned by the Ob-Rb isoform, and this type of isoform activates the JAK-STAT3 pathway. Activation of JAK-STAT3 is important for the nuclear function of leptin, as well as for gene expressions regulating cellular proliferation and anti-apoptosis. The isoform of Ob-Re can be found in circulation. When the JAK-STAT pathway is weakened, leptin still maintains its function in cell proliferation by activating PI3K/AKT, and MAPK pathways [40]. Leptin has become a marker of tumorigenesis in overweight, obesity, and post-menopausal women [40]. Leptin also has the ability to interfere with tamoxifen action in estrogen receptor positive breast cancer cell lines, due to its activation of ERK1/2 and STAT3 signal transduction pathways under estradiol stimulation [41].

Regulation of gene expression and molecular signaling leading to carcinogenesis process in obesity related breast cancers are modified by expression of various microRNA in concert.

## MicroRNA deregulation in obesity, metabolic syndrome, breast cancer

MicroRNAs (miRNA or miR) can modulate expressions of protein-coding genes. Abundant studies showed that miRNA play an important role in adipose metabolism, inflammatory or proliferative signaling as well as in carcinogenesis. There are many miRNAs that have a role in obesity and adipogenesis such as: Let-7, miR-15a, miR-17-92, miR-21, miR-24, miR-27, miR-30, miR-31, miR-103, miR-107, miR-125b, miR-130, miR-138, miR-143, miR-150, miR-200, miR204/211, miR-210, miR-221, miR-222, miR-326, miR-335, miR-355, miR-378,miR-448, and miR-519d [42]. Those miRNAs target adipogenesis process involving various gene expression, transcription factors (PPAR-γ, C/EBP), several signaling pathway (Wnt/catenin, TGF-β superfamily, IGF, and insulin), and extracellular matrix [43]. Several miRNAs may target the same genes however, one single miRNA may also be able to modulate many different target genes playing important role in obesity-related breast cancer.

Some miRNAs regulate adipogenesis by targeting PPAR-γ [43]. MiR-143 known to increase pre-adipocyte differentiation, target PPAR-γ and inhibit ERK5 (Extracellular Regulating Kinase 5) [44]. ERK, a member of MAPK, promotes cell proliferation, angiogenesis, cell differentiation, and survival [44]. ERK5 does not have function in adipogenesis [43], but have prognostic impact in breast cancer [45].

Interaction of PPAR-γ ligand, rosiglitazone, and IGFBP-3 decrease proliferation of breast cancer cells line MCF-7, MDA-MB 468, MDA-MB 231 [46]. PPAR-γ are reported to act as tumor suppressor by regulate proliferation, apoptosis, and cellular differentiation [47]. Overexpression of miR-130 are often found in adipose tissues of obese women along with low expression of PPAR-γ [43] that raised hypothesis that miR-130 suppressed adipogenesis. On the other hand, overexpression of miR-130 inhibit PTEN in human breast cancer cell, and activate AKT phosphorylation [48].

MiR-21 enhanced adipogenesis of human adipose tissue-derived stromal cells (HASC) by modulating TGF-β [49], and targeting STAT3 signaling [50]. Those 2 signaling pathways has interaction with PPAR-γ signaling [47]. Treatment of breast cancer cell with oxidized LDL (mimicking hyperlipidemic condition) will induce inflammation and proliferation signaling mediated by miR-21 overexpression. Overexpression of miR-21 inhibit PTEN and activate AKT phosphorylation [51].

Obesity induce overexpression of miR-24-3p that in turn repress HDL uptake, lipid metabolism, and steroid hormone intake by inhibiting Scavenger Receptor B-1 (SRB1) [52]. Overexpression of miR-24-3p inhibit p27Kip1 [53] and Bim expression therefor increase growth and proliferation of breast cancer [54].

Inflammation induced specific miRNA expression in adipocyte. MiR-155 is overexpressed in obese adipocyte with inflammation state, in line with NFκB. It is probably due its ability to target PPAR-γ [55]. MiR-155 promotes proliferation of breast cancer cells by targeting SOC1 and MMP6 [56].

Mir-210 promotes adipogenesis by suppressing Wnt signaling [57], and in breast cancer it is upregulated by hypoxic condition and target E-cadherin and HIF1-α [58, 59].

Li et al. reported that miR221/222 level are increasing in women with diabetes melitus type2 and postmenopausal breast cancer [60]. MiR221/222 facilitate inflammation in adipocyte tissue and reduce insulin sensitivity by targeting ERα and GLUT4. In breast cancer mir-222 inhibit PTEN, and p27Kip1, activate Akt, inhibit lncRNAGS 5, and MYC [61–66].

Genome‐wide analysis reveals miR‐3184‐5p and miR‐181c‐3p as a critical regulator for adipocytes‐associated breast cancer [67]. Upregulation of miR-3184-5p target FOXP4- NOTCH induced EMT pathway in co-culture of mature adipocyte breast cancer cell. Downregulation of miR-1881c-3p reduce inhibition of PPAR-γ and in turn stimulate breast cancer cells proliferation [67].

MiR-26 targets PTEN/PI3K/Akt to improve insulin sensitivity [68]. MiR-26 acts as a tumor suppressor miR by targeting SLC7A11 [69]. Depletion of miR-26 a/b will increase proliferation of ER-positive breast cancer cell with or without estrogen stimulus. MiR-26 targets estrogen-related genes such as CHD1, GREB1, KPNA2. c-MYC is necessary for inhibiting miR-26 expression induced by estrogen [70].

In chronic inflammation state of obesity, IFN-γ signaling restricts expansion of white adipose tissue (WAT) and decrease insulin sensitivity [71]. miR-30 targets transcription factor STAT1 to limit action of IFN-γ [71]. It also promotes adipocyte differentiation by targeting Plasminogen activator Inhibitor (PAI-1) and Activin Receptor like Kinase 2 (ALK2) [72]. MiR-30a expression in obese adipocyte is repressed. In breast cancer miR-30 inhibits Cyclin E2 result in cell cycle arrest [73].

miR-148-3p targets DNMT1 (a gene involved in DNA methylation) which regulate adipocyte differentiation and obesity [74] and also targets WNT-1/β-catenin, AKT/ERK, IGF-1R signaling pathways in breast cancer [75].

miR-302b maintain SOX2 and c-MYC to produce cytokine-induced cancer stem cell-like properties in breast cancer cell co-cultured with immature adipocyte [76]; whilst miR-302b in breast cancer targets RUNX2, an activator of PI3K/AKT signaling [77].

Let-7 is downregulated in obesity and in vivo it targets HMGA2 [42]. In breast cancer let-7 inhibit HMGA2, MYC, JAK-STAT-3, Caspase-3, RAS, CCND2, ERα [78–80].

Mir-27 are overexpressed in obesity due to hypoxic condition. MiR-27 family blockade PPAR-γ function, activate Wnt1 signaling [81] and in turn suppress GLUT-4 and PI3K-signaling leading to hyperglycemia, insulin resistant, and hyperlipidemia. [82]. MiR-27 in breast cancer act as tumor suppressor miR by targeting SPRY1, BAK, FOXO1, and CBLB/GRB2 [83–85].

MiR-34a overexpressed in visceral fat of overweight/obese subjects are associated with insulin resistant and metabolic inflammation. Lipid loaded mature adipocyte-secreted exosomes transport miR-34a to macrophage and suppress anti-inflammatory M2 phenotype by repressing Kruppel-like factor 4 (Klf-4) [86] Mir-34a is key mediator in exacerbating obesity related systemic inflammation and metabolic dysregulation [86] As contrary, previous studies reported that miR-34a were downregulated in human breast tissue [87]. MiR-34a acts as tumor suppressor miR by downregulating its target genes such as BCL-2 and SIRT1 [88] and Notch1 [89] Wnt/β-catenin signaling pathway [90], fra-1 [91], MYC [92].

Several miRs regulate obesity and breast cancer and their target genes are summarized in Table 1.

**Table 1:** MicroRNA (miR) Deregulation in Obesity and Breast Cancer

MiR	Target genes and functions in obesity	Target genes and functions in breast cancer	References
21	TGF-βR2 inhibition PTEN inhibition, reduced AP-1	PTEN inhibition, PI3K/Akt activation	[49–51]
24-3p	Inhibit SR-B1 that regulate cholesterol uptake, increase HMGCR, DHCR24 and SREBP2	Inhibit p27Kip1, inhibit Bim, cell cycle proliferation	[52–54]
155	Upregulated in inflammation targets PPAR-γ	Downregulate SOC-1, upregulate MMP6	[55, 56]
210	Inhibit Wnt signaling, increase adipogenesis	E-cadherin, HIF1-α, metastasis, proliferation	[57–59]
221/222	Erα, GLUT4, reduced insulin stimulation of glucose uptake	Inhibit PTEN and p27(Kip), activate Akt, ER-alpha, inhibit IncRNA GAS5 down regulate MYC, increase proliferation, cell cycle, survival	[60–66]
3184-3p	FOXP4–NOTCH induced EMT pathway proliferation of MABC cells		[67]
let-7	Inhibit HMGA2, inhibit preadipocyte proliferation	Inhibit HMGA2, MYC, JAK-STAT-3, Caspase-3, RAS, CCND2, Erα decrease invasion tumor suppressor function	[78–80]
26b	Inhibit PTEN/PI3K/AKT pathway to modulate insulin sensitivity	Serpin B2, anti-metastasis and anti-invasion	[68–70]
27b	Control lipid metabolisms inhibit PPAR-γ	FOXO1, ST14 BAK SPRY2 TMEM170B CBLB/GRB2 apoptosis, cell-cycle checkpoint	[81–85]
30a	Suppression of STAT1 to limit Interferon γ-signaling	Cyclin E2; anti proliferative G1, cell cycle arrest	[71–73]
181c-3p	PPARα; reduced inhibition of PPARα, BC proliferation		[67]
143-3p	PPAR-γ, AP2, leptin pathway, ERK5	DNMT3A, PTEN hypermethylation, increase TNFRS F10c methylation, KRAS, AKT1, BCL2	[44]
148a-3p	inhibit DNMT1 which is correlated with obesity	WNT-1, β-catenin, MMP-7, TCF-4, BCl-2, caspases, anti metastasis, anti invasion by regulating Wnt/β-catenin signaling pathway	[74, 75]
302b	Maintain SOX2 and c-Myc by targeting repressor of c-Myc	Target RUNX2, that activate PI3K/AKT signaling and regulate proliferation	[76, 77]
34a	Inhibit macrophage M2 induced adipose inflammation	Inhibit BCL2, CCND1, MYC, E2F3, CDK6, SIRT1 anti-apoptosis	[86–92]

Leptin expressions and functions are also regulated by the orchestration of various miRNA. Stimulation of leptin may modulate several types of miRNAs, both oncogenic and tumor suppressor miRs. Leptin induced oncogenic miRs (miR-21, miR-96, miR-31, miR-182) and reduced tumor suppressor miRs (miR-143, miR-26b, miR-27b, MiR-489) [93]. Increased leptin expression is significantly associated with increase post-menopausal breast cancer risk [93].

Deregulation of some miRNAs in breast cancer are widely documented in various studies. Some of them act as oncogenic miRs that regulate the process of carcinogenesis and metastasis, whilst others act as tumor suppressor miRs that suppress oncogenesis and the proliferation of cancer cells. Some miRNAs expressions are specific for the histologic type of breast cancer [94, 95] Let-7a, let-7b, and miR-324 are specifically upregulated in luminal type breast cancer. MiR-142-5p, miR-155 are downregulated in luminal B type. MiR-106a, miR-18a, miR-155, miR-135b are upregulated in basal type breast cancer. Interestingly, miR-187 is upregulated in HER-2 breast cancer, but miR-130a, miR-30a-3p, miR-30a-5p, and miR-224 are downregulated [95].

It is important to identify deregulated miRNAs in breast cancer patients with obesity or metabolic dysfunction which would have an impact on their prognosis.

**Fig. 1:**
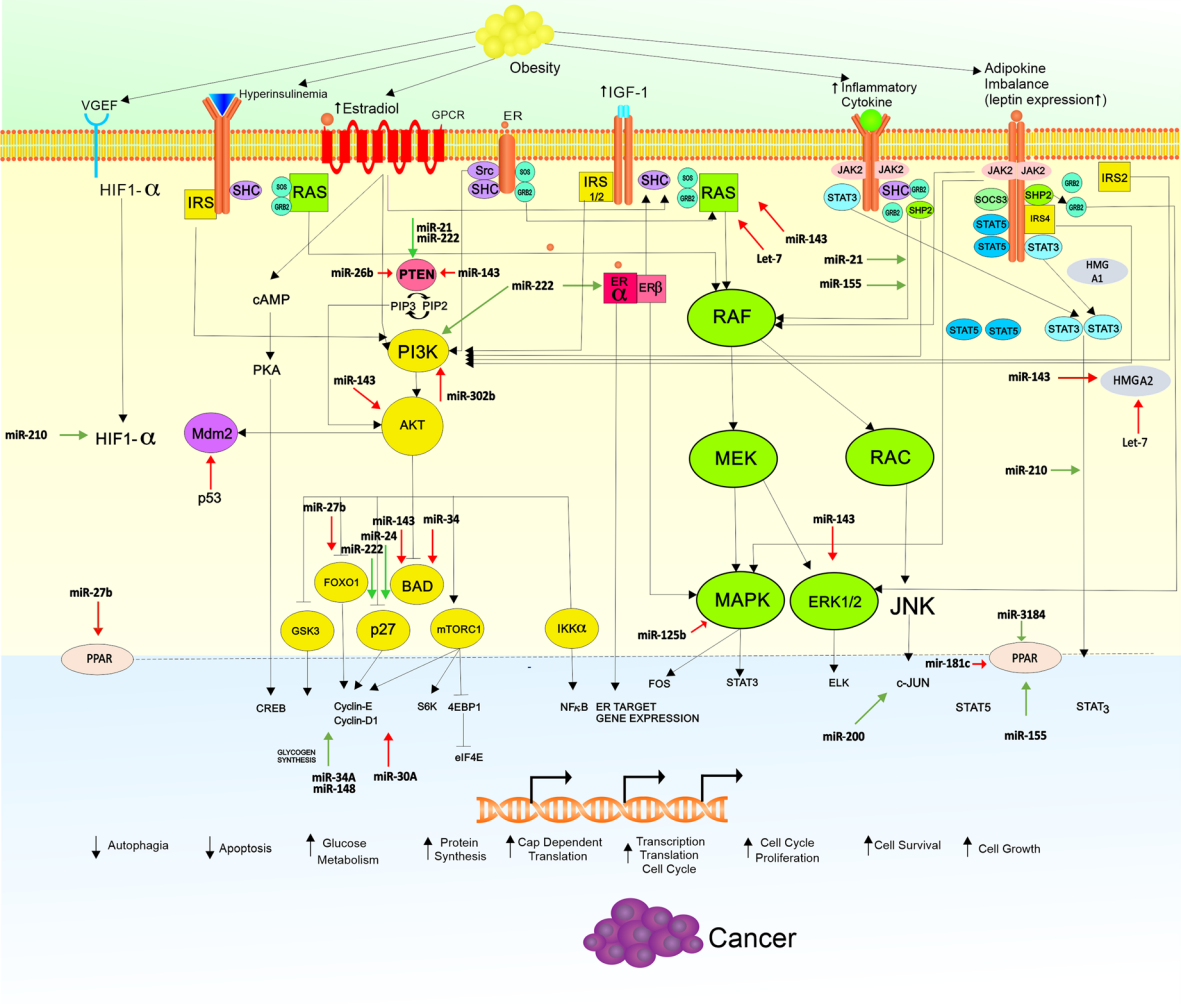
Crosstalk Signaling and miRNA Deregulation in Obesity Related Breast Cancer. Obesity induce hyperinsulinemia, overexpression of leptin, increasing estradiol due to aromatization, Insulin-like Growth Factor 1 (IGF-1) and also Vascular endothelial Growth Factor Receptor (VEGFR) expression. Activation of JAK-STAT pathway, RAS-RAF-MEK-MAPK pathway, PI3K-AKT pathway lead to activation of nuclear transcription that regulate gene expression necessary for suppressing autophagy, apoptosis, and increasing growth, proliferation, cell cycle progression, and cellular survival. Certain microRNAs (miRs) work to regulate expression of protein-coding genes needed in metabolic and carcinogenic process. Green arrow for up-regulation and red arrow for down-regulation. *VEGF* Vascular Endothelial Growth Factor, *GPCR* G protein coupled receptor, *ER* estrogen receptor, *RAS* Rat sarcoma, *RAF* serine threonine protein kinases protooncogene, *MEK/MAP2K1* mitogen activated protein kinase kinase, *ERK* extracellular signal-regulated kinases, *STAT* signal tranducer and activator transcription, *JAK2* Janus Kinase 2, *IRS* insulin receptor substrate, *HIF*-*α* Hypoxia Inducible Factor-α, *CREB* cAMP Response Element-Binding Protein, *cAMP* cyclic Adenosin Monophosphat, *PI3K* phosphoinositide inositol 3 kinases, *AKT* serine/threonine kinas, *Mdm2* mouse double minute 2 homolog, *BAD* Bcl2 associated agonist cell death, *GSK3* Glycogen Synthase Kinase 3, *FOXO* fork head box O, *mTOR* mechanistic target of Rapamycin Kinase, *IκK* I κKinase,. *NFκB* nuclear Factor κB

Figure 1 summarize the crosstalk between metabolic and mitogenic process in obesity related breast cancer and the potential role of some microRNA that regulate both processes.

Some investigators developed miR signatures to evaluate their role as prognostic markers in breast cancer. For example, miR-21, miR-30c, miR-181a, miR-125b, miR-7, miR-200a, miR-135b, miR-22 and miR-200c signatures are tested in hormone positive, HER2- negative breast cancer and provide reliable prognostic models [96]. To date, there was no difference found in miR expressions between obese postmenopausal patients with breast cancer and normal weight groups in terms of miR-17-5p, miR-195-3p and miR-221-3p [97].

On the contrary, there are many miRs upregulated in obesity but acted as tumor suppressor miR (i.e. miR-30, miR-448, and miR-519). According to author personal opinion this might explain some paradoxes found in women with obesity without compromising survival outcome. Several studies showed obesity does not compromise survival outcome in some patients with breast cancer [98–100]. The complexities network of miRs function and regulation make it more difficult to select the signature of miRs as prognostic markers in breast cancer with obesity and metabolic deregulation.

Further exploration is needed to identify certain miR signatures to be developed as a prognostic model in obese/overweight breast cancer.

## Conclusions

Theoretically obesity may induce breast cancer through deregulation of some miRs that regulate the metabolic process, cellular inflammation, and proliferation signaling, pathways via adipokines, insulin-like growth factors, insulin, cytokines, and estrogen signaling. Various miRs are deregulated in patients with breast cancer with co-morbid obesity, suggesting there are some sharing of mechanisms involved in adipogenesis and carcinogenesis. Presently, there is no single miR that can predict prognosis or serve as a single biomarker. Some combination of miR signatures have the potential for a set of prognostic markers specific for the different types of breast cancers, as well as breast cancer with co-morbid obesity, but this possibility needs to be further explored and validated.

## Data Availability

Not applicable.

## Abbreviations

AKT: AKT Serine/Threonine Kinase
BAD: Bcl2 associated agonist cell death
BAK: BCL-2 homologous antagonist killer
BCL2: B cell lymphoma 2
BIM: BCL2L11 protein 11
cAMP: Cyclic Adenosin Monophosphat
CCND1: Cyclin D1 gene
CCND2: Cyclin D2
CCNE2: Cyclin E2 gene
CREB: cAMP Response Element-Binding Protein
DHCR24: 24 Dehydrocholesterol reductase
DNMT2: DNA methyltransferase
E2F3: Transcription factor E2F3
EGFR: Epidermal Growth Factor Receptor
EphA4: Ephrine-type A receptor 4
ER: Estrogen receptor
ERK: Extracellular signal-Regulated Kinases
Foxo1: Fork head box O 1
Fra-1: Fos-related antigen 1
GAS5: Growth Arrest Specific 5
GPCR: G protein coupled receptor
GRb2: Growth factor receptor bound 2
GSK3: Glycogen Synthase Kinase 3
HDAC: Histone deacetylase
HIF1-α: Hypoxia Inducible Factor-α
HMAG2: High motility Group 2
HMGCR: HMG-CoA reductase (3 hydroxy-3 methyl- glutaryl-co enzyme A reductase)
HUR: Human antigen R (Embrionic Lethal, Abnormal Vision Drosophila Like-1 or ELAVL 1)
IGF: Insulin-like Growth Factor
IGF-1R: Insulin-like Growth Factor 1 Receptor
IκK: Iκ Kinase
IRS: Insulin Receptor Substrate
JAK2: Janus Kinase 2
KRAS: Kirsten Rat Sarcoma Virus
Klf-4: Kruppfel-like Factor 4
MAP2K1/MEK1: Mitogen Activated Protein Kinase Kinase
MAP3K2: Mitogen Activated Protein Kinase 2
MdM2: Mouse double minute 2 homolog
MMP6: Matrix Metallo Peptidase-9
MSH2: Muts homolog 2
mTOR: Mechanistic Target Of Rapamycin Kinase
MYB: (myeloblastosis) protooncogene like 1 and Myb related protein B
c-MYC: Cellular MyelocytomatosisNFκB: Nuclear Factor Κb
NFκB: Nuclear Factor κB
P16Ink4a: p16 cyclin dependent kinase inhibitor 2 A
P21/Kip1: CDK inhibitor p21/p27
PI3K: Phosphatidyl inositol-3 kinase
PPAR-γ: Peroxisome Proliferator Activated Receptor γ
PTEN: Phosphatase and tensin homolog
RAS: Rat sarcoma
RAF 1: Rapid accelerated Fibrosarcoma
RUNX1: Runt related transcription factor 3
SIRT1: Sirtuin 1
SLC7A11: Cysteine glutamine transporter
SPRY2: Sprouty homolog 2
SREBP2: Sterol regulatory element binding protein 2
ST14: Suppressor of tumorigenicity 14
STAT: Signal Transducer and Activator Transcription
TIMP3: Tissue Inhibitor of MetalloProteinase-3
TMEM170B: Transmembrane protein 170B
TNC: Tenascin-C gene
TPM1: Tropomyosin gene
WIM/TWF1: Twinfilin Actin Binding Protein
ZEB1, ZEB2: Zinc finger e-box-binding homeobox 1 and 2
